# XVII International AIDS Conference: From Evidence to Action - Regional focus

**DOI:** 10.1186/1758-2652-12-S1-S6

**Published:** 2009-10-06

**Authors:** Parijat Baijal, Rodney Kort

**Affiliations:** 1 Parijat Baijal, Strategic Information Unit, Department of HIV/AIDS, World Health Organization, 1211 Geneva, Switzerland; 2 Kort Consulting, Toronto, M4Y 2T6, Canada

## Abstract

This article summarizes the challenges, opportunities and lessons learned from presentations, discussions and debates addressing major policy and programmatic responses to HIV in six geographical regions: Sub-Saharan Africa, Asia and the Pacific, Eastern Europe and Central Asia, Latin America, Caribbean, and Middle East and North Africa. It draws from AIDS 2008 Leadership and Community Programmes, particularly the six regional sessions, and Global Village activities.

While the epidemiological, cultural and socio-economic contexts in these regions vary considerably, several common, overarching principles and themes emerged. They include: advancing basic human rights, particularly for vulnerable and most at risk populations; ensuring the sustainability of the HIV response through long-term, predictable financing; strengthening health systems; investing in strategic health information; and improving accountability and the involvement of civil society in the response to AIDS.

Equally important is the need to address political barriers to implementing evidence-based interventions such as opioid substitution therapy (OST), needle and syringe programmes (NSPs), comprehensive sexuality education for youth, and sexual and reproductive rights. Finally, these regional discussions emphasized the need for legislative and policy reforms related to structural barriers facing women and girls, MSM, IDUs, sex workers and migrant populations.

## Discussion

### Sub-Saharan Africa

Sub-Saharan Africa remains the region most heavily affected by the epidemic, accounting for 67% of all PLHIV and nearly 75% of all AIDS deaths in 2007. Almost 90% of the children with HIV worldwide live in sub-Saharan Africa, and HIV is the underlying cause of almost one-third of all child deaths in some high-burden countries [1].

South Africa has the world's largest national epidemic, with an estimated 5.7 million PLHIV. Approximately 500,000 people are newly infected each year and 1,000 people die every day from AIDS-related illnesses. Young women in South Africa face a substantially greater risk of becoming infected than men [2].

22 million people live with HIV in sub-Saharan Africa. 1.5 million people died from AIDS-related causes in 2007. 1.9 million people were newly infected in 2007. The most vulnerable populations include women and girls, children and youth.

#### Lessons learned

The regional session on sub-Saharan Africa acknowledged the tremendous progress in national responses to AIDS in the past few years, including substantial increases in ART coverage [3]. Declining HIV prevalence has been observed in several countries, particularly in the smaller HIV epidemic of West Africa. In his overview of the regional response, Kaptue (Université des Montagnes, Cameroon) praised the achievements of countries such as Botswana, Kenya, Malawi, Namibia and Zambia, where political commitment, multisectoral coordination and the active participation of civil society have shown success despite limited resources [4]. However, stabilizing or declining HIV prevalence may at least partially be due to approximate parity between AIDS mortality and new infections, raising questions about the extent of the efficacy of prevention interventions. Countries facing civil war and/or severe economic and political crisis have produced data suggesting that HIV is on the decline when the opposite may in fact be occurring. As such, speakers noted that recent HIV prevention successes cited by UNAIDS in Zimbabwe should be viewed with caution, as emigration and dispersal of HIV-positive people may mask actual HIV prevalence rates. A late-breaker study addressing the impact of conflict - specifically post-election civil unrest in Kenya - revealed a significant negative impact on ART adherence and other health outcomes [5].

In Tanzania, the President and First Lady launched a national HIV testing and counselling campaign in July 2007 by taking a voluntary HIV test. By April 2008, the number of people who took an HIV test was nearly 10 times the total average number of people who take a test in a year. Namibia's recent Demographic and Health Survey reported one of the highest rates of HIV testing in the region [6].

In Cameroon, where the national antiretroviral therapy programme has been implemented with progressive decentralization, a study concluded that district-level HIV services performed as well as the central and provincial services [7]. A study in Swaziland found that providing community-based antiretroviral therapy reduced the number of missed appointments among patients [8].

**Figure 1:**
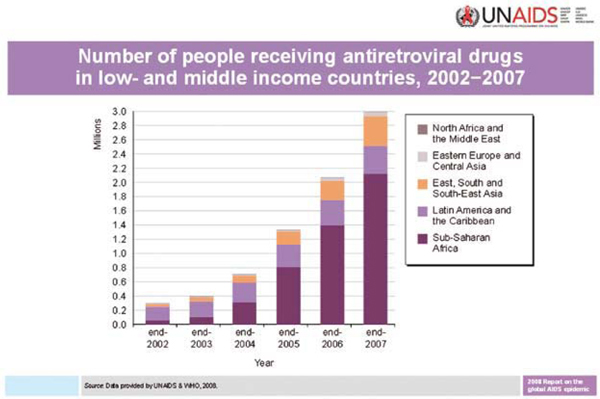
**UNAIDS number of people receiving antiretroviral drugs in low and middle income countries, 2002-2007**.

In 2005 the Botswana government contracted a private management company to outsource antiretroviral therapy provision to the private health sector and ease congestion in public facilities. By May 2007, almost 6,000 patients had been enrolled in antiretroviral therapy and the waiting lists in the public sector reduced dramatically [9]. Also in Botswana, more than 90% of HIV-positive pregnant women received antiretrovirals in 2007 to prevent transmission to their children [10]. The increase in ART coverage is outlined in Figure 1.

Other speakers cautioned against complacency in this region's response to HIV/AIDS. Elizabeth Mataka, UN Special Envoy for AIDS in Africa, called for raising awareness on the risks of multiple, concurrent partnerships, and noted the resurgence in sexual risk behaviour in countries such as Uganda, where the epidemic had begun to stabilize [11]. The report-back from the 11^th ^meeting of the Society for Women against AIDS in Africa (SWAA) by Soulymane Mboup noted that, while there had been significant improvement in mainstreaming gender issues into policies and plans, implementation has been largely unaffected; promoting the sexual, reproductive, and human rights of women infected and affected by HIV/AIDS and addressing gender-based violence will be integral in effectively addressing gender equity issues and - ultimately - the overall response to AIDS in this region [12].

#### Challenges and opportunities

HIV programmes in sub-Saharan Africa continue to face a critical shortage of health workers and health care infrastructure. Speakers from Uganda and Malawi - countries which have both made substantial progress in expanding their HIV response in recent years - described how the "second wave" of scale-up will not be sustainable without building human resource capacity and providing better working conditions and other incentives aimed at staff retention [13]. They also emphasized the need to strengthen infrastructure at all levels of the health care delivery system, outreach to rural areas and hard-to-reach populations, and integrating HIV services within the public health care delivery system, an issue that, as session Co-Chair Bience Gawanas noted, is complicated by the enormous challenges posed by the dual epidemic of TB and HIV.

Advocates emphasized that respect and recognition of human rights, irrespective of gender, sexual orientation or ethnicity, must become central to the response in the region. Mataka and other speakers argued that HIV programmes in sub-Saharan Africa have largely neglected the needs of men who have sex with men, held back by denial and homophobia, as well as the needs of IDUs. While political leaders at the conference expressed support for addressing the needs of these groups, future conferences will judge whether the rhetoric will translate into badly needed legal and policy reforms. To date, the actions of national governments - from the homophobic statements of the President of Gambia (who recently threatened to kill all homosexuals) to the arrest of three Ugandan gay activists at the June 2008 HIV/AIDS Implementers' Meeting - seem to more accurately reflect the position of many policymakers in this region [14,15].

The importance of sound strategic health information was also raised during a number of sessions, both as the basis for designing and improving programmes, as well as a gauge of transparency and accountability. Estimates of the size of populations at high risk of infection (such as MSM and IDUs) are limited, and data on coverage of services among these groups were absent from many country reports. Speakers noted that community-based organizations can play a greater role in monitoring progress and holding governments accountable at future conferences, but to do so must have access to greater technical support and capacity to undertake "shadow-reporting" alongside official government reports.

While substantial progress has been made in scaling up priority HIV interventions in sub-Saharan Africa, that progress remains fragile and more attention and resources are required to address both health system capacity and the needs of vulnerable and most at risk populations.

### Asia and the Pacific

The HIV epidemic in Asia and the Pacific is one of the most diverse in the world, with epidemiological trends varying widely depending on the country and sub-region. Epidemics in Cambodia, Thailand and Myanmar show declining HIV prevalence, while those in Viet Nam, Indonesia and Papua New Guinea are growing. New infections are also increasing in populous countries such as China and Bangladesh.

Five million people live with HIV in Asia, 74,000 in the Pacific. 380,000 people died from AIDS related causes in 2007 in Asia, 1,000 in the Pacific. 380,000 people were newly infected with HIV in 2007 in Asia, 1,000 in the Pacific. The populations most at risk include sex workers, IDUs and MSM.

#### Lessons learned

Countries such as Thailand, Cambodia and some Indian states were widely recognized for their effective and focused HIV responses, especially their campaigns encouraging 100% condom use in sex work settings. In Tamil Nadu, India, a programme targeting sex workers introduced in 1995 under a tripartite agreement between government, community organizations and funding agencies has resulted in a dramatic increase in condom use among female sex workers and truckers [16].

In Malaysia, the government allowed the introduction of harm reduction programmes in 2005, and by 2008, there were more than 22,000 drug users on opioid substitution therapy, with more than one million needles distributed, and a methadone programme introduced in prisons [17]. In China, Viet Nam and Indonesia, the response has recently begun to gather pace and has shown moderate success. Harm reduction programmes for injecting drug users are gradually expanding in the region; however, men who have sex with men have been largely overlooked.

Wimonsate (Ministry of Public Health, Thailand) reported rapidly growing rates of HIV incidence among MSM in a cohort from Bangkok. 1,000 HIV-negative MSM were recruited and tested for HIV every four months. To date in the on-going study, the annual HIV incidence in the group is estimated at 5.1% [18].

In Bangladesh, information on HIV prevention was integrated into the school education curriculum, taking into the account the local cultural and religious context [19]. Papua New Guinea faces a rapidly expanding epidemic in a context of weak health infrastructure, weak political commitment, and high rates of violence against women. While there have been some recent achievements in the national response, effective advocacy is constrained by the challenges of mobilizing civil society, involving PLHIV, and the geographical and linguistic heterogeneity of the country.

**Figure 2:**
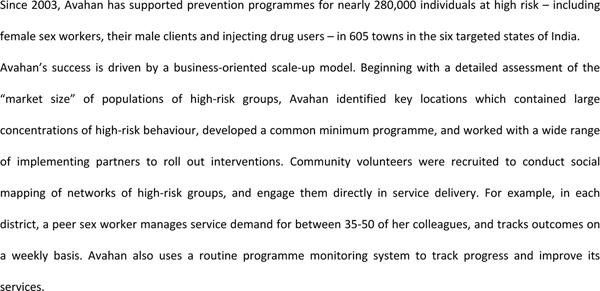
**The Avahan initiative**.

The Avahan initiative was launched in India by the Bill & Melinda Gates Foundation in 2003, with the objective of increasing access to HIV prevention in six states with India's highest HIV prevalence rates. Working with the national, local and district governments and major nongovernmental organizations, Avahan provides funding and technical support to distribute condoms, provide screening and treatment for sexually transmitted infections, and expand peer outreach within key populations (see Figure 2) [20].

#### Challenges and opportunities

Recommendations from the independent Commission on AIDS in Asia emphasized the urgent need to focus on populations most at risk, and called on political leaders to increase their knowledge of the dynamics of the epidemic in their countries and to invest in evidence-based interventions [21]. The recommendations were echoed by speakers at the regional session on Asia and the Pacific [22].

**Figure 3:**
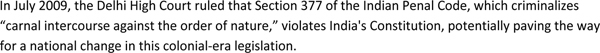
**The Delhi High Court Decision**.

Several sessions also highlighted a number of cases where laws that criminalize drug use, sex work and homosexuality continue to obstruct HIV service provision. In Thailand and Myanmar, for example, drug users continue to face incarceration and even death at the hands of law enforcement officers [23]. In Cambodia and China, sex workers face violence and human rights violations [24]. Sex between men remains illegal in most of Asia and is therefore driven underground. Some examples of progress are emerging: in India, for instance, the Minister of Health expressed support at the conference for changing the Indian Penal Code, which criminalizes homosexuality [25]. However it is clear that a stronger commitment from political leadership is needed to ensure equal access to HIV services for these groups (Figure 3).

Other priorities in the region include expanding access to treatment and care, including affordable second-line drugs; treatment adherence; strengthening links with TB and HCV programmes; and integrating HIV services with maternal and reproductive health services. Increasing the uptake of HIV testing and counselling services is key, as is protecting the privacy and confidentiality of people accessing those services.

The need for reliable data on the populations affected by HIV and their access to services was also raised by speakers. Greater investment is needed not only in generating data to evaluate and improve programmes, but in building capacity to analyse and use data from different sources. The extensive work undertaken by the Commission on AIDS in Asia in reviewing existing evidence and developing recommendations will serve as the basis for expanding the response to the epidemic in the region, and for assessing progress at future conferences.

### Eastern Europe and Central Asia

This region includes one of the fastest-growing HIV epidemics, particularly in the Russian Federation and Ukraine, and is concentrated primarily among IDUs, with significant overlap between injecting drug use and sex work. While political attention and resources allocated to HIV are growing, the policy and programmatic response - including government efforts to include civil society groups in decision-making - has been uncoordinated and inconsistent.

By the end of 2007, 1.5 million people were living with HIV in Eastern Europe and Central Asia, 58,000 had died from AIDS-related causes and 110,000 people were newly infected. Most at risk populations include IDU, sex workers and MSM.

#### Lessons learned

In the Russian Federation, domestic resources allocated to the HIV response have increased fifty-seven fold from 2005 to 2007 (up to 10.7 billion roubles or US$444.8 million in total), based on Russia's 2008 Country Progress Report [26]. In Uzbekistan, IDU "trust points", which offer needle and syringe programmes and substitution therapy, are slowly being scaled up [27]. In Moldova, harm reduction interventions are being expanded in prison settings [28].

Ukraine has the highest estimated HIV prevalence in the region, with very high infection levels among IDUs. The Ukrainian government has demonstrated strong political commitment to address the epidemic, and access to substitution therapy and antiretroviral therapy for injecting drug users is slowly scaling up. The first pilot substitution therapy project was introduced in Ukraine in 2004, and had expanded to 11 sites by 2007. A national operational plan to scale up opioid substitution therapy in Ukraine between 2007 and 2011 is being finalized [29].

The number of people receiving ART in the Ukraine expanded from 137 in 2004 to nearly 8,000 people in 2007 after stewardship of a grant from the Global Fund was transferred from the Ministry of Health to the HIV/AIDS Alliance, due to problems with grant management by the government recipient. The Alliance, working in close collaboration with the Ministry of Health, the Ukrainian AIDS Centre and the All-Ukrainian Network of People Living with HIV/AIDS, has demonstrated how a multidisciplinary approach with shared responsibility is successful in scaling up service delivery at a national level.

#### Challenges and opportunities

Universal access to HIV prevention, treatment and care in Eastern Europe and Central Asia will not be achieved unless policy and legislative changes take place that decriminalize homosexuality and sex work, and increase evidence-based drug treatment and prevention services for IDUs (including making buprenorphrine and methadone available as OSTs). The overall access to these services for IDUs remains unacceptably low.

**Figure 4:**
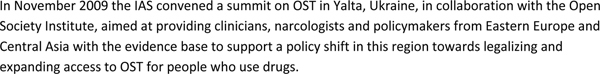
**The Yalta summit**.

Although access to prevention and treatment services for IDUs are expanding, many projects are still in the pilot phase. Consistent access to NSPs remains limited, and some countries, including the Russian Federation, still do not provide OST. At the conference Global Fund Executive Director Michel Kazatchkine called on the international community to engage in dialogue with the Russian Federation on the effectiveness of harm reduction interventions, including OST (Figure 4)[30].

Data on the epidemic among MSM is very limited, although evidence suggests that the epidemic among this population may be substantially larger than official figures estimate. The Ukrainian HIV surveillance system, for example, only reported 159 HIV transmissions from sex between men since reporting began, even though there are an estimated 40,000 MSM living with HIV in the country [31]. The region has made better progress in PMTCT, with coverage at 71% in 2007. Access to antiretroviral therapy is also increasing, although coverage was only 17% in 2007, and drug prices remain high. Co-infection with TB and hepatitis B and C are highly prevalent in the region [32].

At the conference speakers advocated for improved HIV surveillance and monitoring, including a disaggregation of service indicators by sex and risk group, and monitoring of treatment outcomes and drug resistance. Participants also cautioned that while the epidemics in the region were categorized as concentrated, large numbers of people were infected, drawing attention to the probability of an underestimation of the burden of disease, especially among MSM, sex workers and prisoners.

Speakers also called for integrating HIV prevention and care within overall health systems strengthening and service quality assurance measures in these countries. Civil society representatives also called for greater cooperation between civil society organizations and government agencies in policy and decision-making processes, which to date has been very limited [33].

### Latin America

Having Mexico host AIDS 2008 meant that regional response to HIV was at the centre of discussion and debate. Local AIDS 2008 Co-Chair Luís Soto-Ramírez noted that, "the conference has had an enormous impact in Mexico and throughout Latin America. People here are now talking openly about HIV and AIDS. It is really going to help not only men who have sex with men, sex workers and drug users but also migrants and indigenous communities in Mexico" [34]. Significant numbers of new infections continue to occur among MSM, sex workers and, to a lesser extent, IDUs. An estimated 200,000 people are living with HIV in Mexico alone.

1.7 million people live with HIV in Latin America. 63,000 people died from AIDS-related causes in 2007. 140,000 people were newly infected in 2007. The populations most at risk for HIV include MSM and sex workers.

#### Lessons learned

AIDS 2008 provided an opportune platform to bring Latin American issues to the forefront. Immediately prior to the start of the conference, the 1st International March against Stigma, Discrimination and Homophobia was held in Mexico City and Latin American and Caribbean Ministers of Education and Health signed a declaration pledging to implement comprehensive sex education and sexual health promotion programmes among young people [35]. At the conference, the Director of CENSIDA, Mexico's national HIV programme, Jorge Saavedra presented a plenary focused on sex between men [36]. The founder of the Argentine Association of Female Sex Workers presented a plenary address focusing on issues related to sex work, gender inequality, sexual violence and labour rights [37]. The President of Panama, the last Latin American country criminalizing homosexuality, announced during the conference he had signed an executive repealing the legislation.

Brazil, widely acknowledged for its successful ART programme, has also taken steps to protect the rights of sex workers. Sex work is not a crime in Brazil, and the government promotes HIV prevention education and self-esteem among sex workers, including a high profile communications campaign with the tagline, "no shame girl, you're a professional" [38]. RedTraSex, the Latin American and Caribbean Network of Female Sex Workers, supports member organizations in the region to conduct peer outreach and promote sex workers rights. In Ecuador, sex workers recently obtained the right to carry the same healthcare card as everyone else, guaranteeing the same access to essential health services as all women [39]. In Peru, a research project in Lima is using new approaches to reach out to MSM and women to promote HIV prevention and care [40].

### Mexico's response

The epidemic in Mexico is concentrated among MSM and sex workers and is growing among IDUs. In response, the government and nongovernmental organizations have undertaken several prevention efforts among sex workers and gay and other MSM. Mexico City has gone even farther in addressing discrimination against gay/MSM by legalizing gay marriage, one of the first jurisdictions to do so in the region, and implementing an HIV awareness campaign to coincide with AIDS 2008. Mexican civil society groups have been actively engaged in collaborating on these issues with government, and Mexico City Mayor Marcelo Ebrard specifically spoke out against discrimination and homophobia during his speech at the Closing Session.

Antiretroviral therapy, provided free of charge in the public sector since 2003, covered about 57% of estimated need in Mexico at the end of 2007 [41]. During the conference, Mexican President Felipe Calderón announced the repeal of a policy which prevented foreign pharmaceutical companies from selling antiretrovirals in the country unless they were manufactured there [42]. Needle and syringe programmes have expanded, and the Mexican government also agreed to provide participants who use drugs with methadone during the conference, even though methadone is illegal under Mexican law [43].

Under the leadership of Jorge Saavedra, Mexico's first openly gay senior government official, the country has taken a number of steps to address homophobia and discrimination against MSM and to improve their access to health services. Overall spending on HIV programmes increased substantially in Mexico between 2001 and 2005, including funding for programmes targeting MSM. In 2005, Mexico launched a government-endorsed nationwide anti-homophobia campaign, and is now working towards declaring the 51 new HIV ambulatory care clinics in Mexico as "homophobia-free services" [44].

#### Challenges and opportunities

In an overview of the regional response, Cesar Nunez from UNAIDS called for scaling up the regional response with sustained political leadership, resource mobilization, and greater involvement of civil society to fight stigma, discrimination and homophobia [45].

Despite progress in many countries, sex workers and MSM continue to face denial, exclusion and violence. The Latin America and Caribbean Sex Worker Network reported that 34 sex workers in Latin America were killed in the ten months preceding the conference [46]. Speakers also called for greater attention to the needs of indigenous people and migrant workers [47].

While several countries have made progress in scaling up access to treatment and care for people living with HIV, the region still faces the challenge of limited trained human resources and inadequate health infrastructure, especially in rural areas. Sustainable financing is necessary, both from domestic and international sources; one of the financing issues faced by several countries in this region is their current ineligibility for Global Fund grants.

Many speakers also emphasized the need for better strategic health information on the epidemic. There is limited data on the dynamics and epidemiological trends among populations at high risk, and little is known about their access to HIV services. Many countries in the region do not have national monitoring and evaluation plans for the HIV response, which compromises both decision-making and accountability [48]. Greater capacity to interpret and use data, including among civil society organizations, is also necessary to strengthen programmes and assess outcomes.

### The Caribbean

The Caribbean has the highest HIV prevalence of any region outside sub-Saharan Africa, with approximately 75% of Caribbeans living with HIV residing in Haiti (the poorest country in the Western hemisphere) or the Dominican Republic. Overall prevalence in the region is approximately 1% [49]. Poverty, unemployment, early initiation of sexual activity and lack of HIV awareness are all contributing to new infections in this region, among which over 50% are women [50,51].

In 2007, 230,000 people were living with HIV in the Caribbean, there were 14,000 AIDS-related deaths and 20,000 new HIV infections; sex workers, MSM and women are the populations at highest risk of infection.

#### Lessons learned

In 2001, Caribbean countries adopted the Caribbean Regional Strategic Framework for HIV/AIDS, a coordinated, regional approach to addressing the HIV epidemic. Many have developed national strategic plans and legislation, and have expanded HIV services. Speakers at AIDS 2008 reaffirmed their commitment to the strategy and to their national and regional plans. Public health authorities also committed to eliminate mother to child transmission of HIV and syphilis in the Caribbean by 2015 [52].

There has been substantial progress in scaling up access to antiretroviral therapy in countries such as Cuba, Barbados and Jamaica, as well as a decline in rates of mother-to-child transmission [53]. However, there is little epidemiological data for most at risk populations, largely because of intense stigma and the resulting difficulties in reaching these groups.

#### Community-based strategies in Haiti

Claude Pean from Haiti presented the experience of the Fame Pereo Institute, which provides HIV education, testing and counseling, prevention of mother-to-child transmission, treatment and support through mobile teams of health workers who approach low-income communities in households, schools, churches and other public places [54]. This community-based approach is being used to scale up access to ART in Haiti. A study conducted in clinics managed by Partners in Health concluded that task-shifting using a nurse-centred approach to HIV care in rural Haiti was an effective model of scale-up, and resulted in good clinical outcomes for patients [55]. Another Haitian study found that regular support and follow-up by community health workers helped to improve retention in HIV care [56].

#### Challenges and opportunities

Many speakers emphasized the imperative of addressing homophobia, HIV-related stigma and discrimination in the Caribbean, and decriminalizing sex work and sex between men if countries are to achieve universal access. Without a change in public attitudes and relevant legislation, populations at high risk for HIV infection will continue to face barriers in accessing basic health care. Speakers also noted the critical importance of addressing the needs of young people and finding ways to engage them in HIV prevention and control efforts.

Participants also agreed on the importance of strengthening HIV surveillance and monitoring in the region, especially among populations at highest risk for HIV infection, and on the need to address broader developmental issues as an important precursor to expanding access to services. Many countries in the Caribbean are limited by poor public sector infrastructure and fragile economies, and poverty and unemployment contribute to the rise of transactional sex, population mobility and gender inequality.

#### Middle East and North Africa

At the Regional Session on the Middle East and North Africa, Tawil (UNAIDS, Switzerland) emphasized that there is no single HIV epidemic in this region, and that the response has been shaped by diverse socio-political and epidemiological contexts [57]. He described the regional response in three overlapping categories: a "comprehensive" response in countries such as Djibouti, Iran, Morocco, Somalia and Sudan; an "adaptive and potentially effective" response in countries such as Jordan, Lebanon, Tunisia and Yemen; and responses "limited" either by political constraints (such as Egypt and Libya), or by war or post-war contexts (Afghanistan, Iraq, Occupied Palestinian Territories).

In 2007, 380,000 people were living with HIV in the Middle East and North Africa, there were 27,000 AIDS-related deaths and 40,000 new HIV infections in the region. The populations most at risk for HIV are sex workers, IDUs and MSM.

#### Lessons learned

An Algerian speaker emphasized the importance of involving PLHIV in the national response and developing their capacity to participate in planning and implementation, including efforts to fight stigma and build links with religious leaders [58]. She highlighted the importance of the Algiers Declaration of People Living with HIV, adopted in 2005, which brought together people living with HIV from different countries in the region, and the subsequent establishment of support groups in many countries, including the Islamic Republic of Iran, Morocco, Tunisia and Yemen.

**Figure 5:**
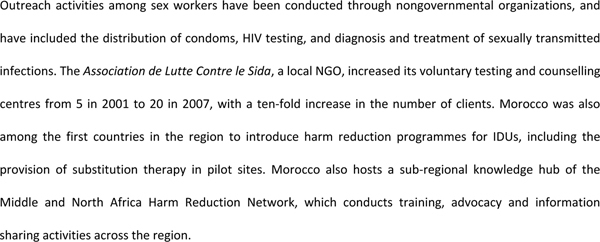
**The Moroccan Example**.

Amal Karaouaoui of Morocco presented perspectives from the field on providing HIV prevention services to populations at high risk [59]. She noted that while homosexuality and sex work lie within a "forbidden zone" in the socio-political and religious context of the region, a number of countries were using community approaches to reach out to risk groups (Figure 5). In the Sultanate of Oman, community workers approach sex workers and MSM, engage them in awareness efforts, and create a bridge between these vulnerable groups and health services. In Sudan, outreach workers disseminate health information among tea-sellers and sex workers. She called on other countries in the region to follow these examples, acknowledging both the difficulty and promise inherent in the work.

Other speakers noted the achievements in the Islamic Republic of Iran, where prevention and treatment services for IDUs are provided in 600 addiction clinics in the country and more than a 100,000 IDUs are receiving OST, including inmates of prisons [60].

### Challenges and opportunities

The main challenge in scaling up the HIV response in North Africa and the Middle East remains the social stigma and marginalization faced by the population groups at high risk. Access to health information among high risk groups is very limited, as is their access to health, social and legal services. Effective outreach must be linked with facilitating their access to HIV testing, treatment and care, and dispelling stigma and taboos. Many speakers also called for expanding dialogue with political and religious leaders and building the capacity of civil society organizations for more effective outreach to such groups.

Participants also raised the need to strengthen surveillance and monitoring in the region. Little is known about the nature and scope of the epidemic among high risk groups, due to both inadequate surveillance systems and the stigma associated with these groups. Community based organizations, which play a crucial role in providing outreach to marginalized populations in the region, can also play a greater role in monitoring their access to services and health outcomes. This will require greater investments in capacity-building to equip them with the necessary skills and resources.

## Conclusion

The conference raised a number of policy and advocacy issues that must be addressed to achieve universal access to HIV prevention, treatment and care. While some issues are specific to the epidemiological and socio-economic context in a particular country or a region, a consensus emerged on several overarching themes that must be addressed in the scale-up of national HIV responses:

### 1. Advance universal human rights

The need to address protect the human rights of women and girls, gay and other MSM, IDUs and sex workers in country-level AIDS planning was echoed repeatedly in a number of the country and regional presentations at AIDS 2008. National governments must acknowledge the needs of the groups most affected by HIV, expand their access to health services, and most importantly, remove legal and policy barriers to scale up by decriminalizing sex work and homosexuality, taking concrete measures to address the root causes of gender inequality, and embracing the implementation of effective harm reduction strategies for IDUs, including NSPs and OST. Governments must also build more meaningful collaboration with civil society groups, especially groups representing people living with HIV, in the development, implementation and monitoring of programmes. The early mantra of AIDS activism, "nothing about us, without us", became a common theme at the conference.

### 2. Sustainability and strengthening health systems

Many countries in resource-limited settings stressed the need to strengthen human resource capacity and provide training and incentives for health workers to scale up the HIV response. Strategies such as task-shifting, which is showing positive outcomes, must be rolled out with due attention to ensuring service quality and protecting patient rights. Sustained scale-up of HIV programmes also requires adequate health infrastructure, including drug procurement and supply systems, laboratories, and service provision to rural and other hard to reach populations. Presentations throughout the conference echoed the need for building synergies between HIV prevention and treatment programmes and other health services. Advocates also called for the removal of ceilings on health expenditure imposed by the International Monetary Fund, and called on countries to step up their own domestic expenditures on health. A sustained response to HIV will also rely on continued financial support from the international community, including the Global Fund to Fight AIDS, TB and Malaria.

### 3. Invest in strategic health information and improve accountability

There was clear consensus on the need for more robust data to understand the burden and trends of the epidemic, to inform programme development, and to measure outcomes. The lack of data regarding populations at high risk of HIV, especially MSM, was evident in all country sessions. The health needs of these groups will continue to be neglected unless countries commit to knowing the size of populations at risk, monitoring disease trends, and tracking their access to services. It was also apparent from many sessions that data needs to be interpreted with caution; in a number of country presentations, ART coverage was measured only against the number of people who know their HIV status and are enrolled in public services, overlooking the many who are undiagnosed. The international community must play its part to ensure that accurate, complete data are being collected and reported by countries. Civil society organizations are increasingly willing to build their own capacity to monitor progress and hold governments and international agencies accountable.

## Competing interests

Parijat Baijal is employed with the World Health Organization and Rodney Kort is an independent consultant contracted by the International AIDS Society for the purpose of preparing and editing the AIDS 2008 Impact Report for publication.

## Authors' contributions

Parijat Baijal drafted the initial text and Rodney Kort provided editorial input and advice. Both authors have approved the manuscript for publication.

## References

[B1] UNAIDS/WHO Report on the global AIDS epidemic 2008 Geneva 2008

[B2] UNAIDS/WHO Report on the global AIDS epidemic 2008 Geneva 2008

[B3] Regional Session on sub-Saharan Africa Symposium, XVII International AIDS Conference, August 2008, MOSY04

[B4] Kaptue L Status of the response in the region - regional overview Symposium, XVII International AIDS Conference, August 2008, MOSY0401

[B5] Vreman R The impact of the Kenya post-election conflict on medication and visit adherence for HIV-infected children in Western Kenya Abstract Session, XVII International AIDS Conference, August 2008, THAE0401

[B6] Shein AM Regional session on sub-Saharan Africa Session. XVII International AIDS Conference, August 2008, MOSY04

[B7] Protopopescu C Health-related quality of life among ART-treated HIV-infected patients in Cameroon: the impact of HIV services decentralization Poster Exhibition, XVII International AIDS Conference, August 2008, THPE0828

[B8] Humphreys C Effectiveness and safety of nurse-led primary care based antiretroviral treatment in a resource constrained setting Abstract Session, XVII International AIDS Conference, August 2008, WEAB0206

[B9] Puvimanasinghe JPA Evaluation of the outsourcing of ART services to the private sector in Botswana XVII International AIDS Conference, August 2008, MOPE0996

[B10] Gaolathe T Mother-to-child HIV transmission rate in Botswana - analysis of dried blood spot (DBS) results from the national PMTCT programme Abstract Session, XVII International AIDS Conference, August 2008, THAC0402

[B11] Mataka E Regional session on sub-Saharan Africa Symposium, XVII International AIDS Conference, August 2008, MOSY04

[B12] Mboup S Key lessons from the latest regional AIDS conferences Symposium, XVII International AIDS Conference, August 2008, MOSY0402

[B13] Presentations at the session on Universal ARV Scale Up: Delivering the Second Wave Special Session, XVII International AIDS Conference, August 2008, WEBS02

[B14] President plans to kill off every living homosexual allAfrik.com http://en.afrik.com/article13630.html

[B15] Gay arrests in Uganda condemned 2008 BBC News http://news.bbc.co.uk/2/hi/africa/7437854.stm

[B16] Krisostomo V Key learnings and lessons from Asia Pacific Symposium, XVII International AIDS Conference, August 2008, MOSY1004

[B17] Kamarulzaman A Substance Use and Harm Reduction Plenary Session, XVII International AIDS Conference, August 2008, TUPL0102

[B18] Wimonsate W Successful start of a preparatory HIV cohort study among MSM in Bangkok, Thailand Abstract Session, XVII International AIDS conference, August 2008, MOAC01

[B19] Bosu AK Integrating sustainable HIV prevention information into Bangladesh's national curricula - a best practice case from the South Asia region Poster Discussion, XVII International AIDS Conference, August 2008, TUPDC202

[B20] The Avahan India project was presented at a number of sessions at the conference, including Leading the AIDS Response in Asia: the report of the Commission on AIDS in Asia Satellite Session, XVII International AIDS Conference, August 2008 TUSAT16; and New Frontiers in HIV Prevention Science. Symposium, XVII International AIDS Conference, August 2008, TUSY08

[B21] Commission on AIDS in Asia: genuine community consultation influencing regional recommendations Poster Exhibition, XVII International AIDS Conference, August 2008, MOPE0926

[B22] Regional Session on Asia and the Pacific Symposium, XVII International AIDS Conference, August 2008 MOSY10

[B23] Kamarulzaman A Substance Use and Harm Reduction Plenary Session, XVII International AIDS Conference, August 2008, TUPL0102

[B24] Reynaga E Sex Work Plenary Session, XVII International AIDS Conference, August 2008, WEPL0103

[B25] The Hindustan Times 2008 http://www.hindustantimes.com/StoryPage/StoryPage.aspx?sectionName=&id=104e918a-9a93-4879-9893-1861d0279c56&&Headline=Gays+upbeat+over+Ramadoss'+support&strParent=strParentID

[B26] Country Progress Report of the Russian Federation on the Implementation of the UNGASS Declaration of Commitment on HIV/AIDS, January 2006-December 2007 Ministry of Health and Social Development of the Russian Federation 2008

[B27] Makhkamov A Trust points as an effective approach to scale up HIV prevention among injecting drug users Abstract Session, XVII International AIDS Conference, August 2008, CDB0334

[B28] Gherman L Comprehensive approach to HIV prevention in Moldova prison settings Abstract Session, XVII International AIDS Conference, August 2008, CDC0549

[B29] Klepikov A The progress in Ukraine Bridging Session, XVII International AIDS Conference, August 2008, WEBS0203; and Universal ARV Scale Up: Delivering the Second Wave. Bridging Session, XVII International AIDS Conference, August 2008, WEBS02

[B30] Regional Session on Eastern Europe and Central Asia Symposium, XVII International AIDS Conference 2008, WESY04

[B31] Kis Z HIV prevention, care and support for MSM in a post-Soviet country: fighting with old stereotypes Symposium, XVII International AIDS Conference, August 2008, WESY0503

[B32] WHO, UNAIDS and UNICEF Towards universal access scaling up priority HIV/AIDS interventions in the health sector: Progress Report 2008 2008 WHO http://www.aids2008.org/admin/images/upload/829.pdf

[B33] Kizub D Personal Communication to Ron MacInnis 2008

[B34] Flynn M AIDS 2008: Final comments from Conference Chairs and IAS President http://www.aids2008.org/admin/images/upload/829.pdf

[B35] Leaders pledge to promote sexual health to stop HIV in Latin America and the Caribbean UNAIDS http://www.unaids.org/en/KnowledgeCentre/Resources/FeatureStories/archive/2008/20080731_Leaders_Ministerial.asp

[B36] Saavedra J Sex between men Plenary Session, XVII International AIDS Conference, August 2008, TUPL0104

[B37] Reynaga E Sex Work Plenary Session, XVII International AIDS Conference, August 2008, WEPL0103

[B38] Batista Simao M Brazilian Response to AIDS in mobilising community based response Satellite Session, XVII International AIDS Conference, August 2008, SUSAT4402

[B39] Reynaga E Sex Work Plenary Session, XVII International AIDS Conference, August 2008, WEPL0103

[B40] Caceres C Vulnerable men who have sex with men and women in Lima: feasibility of new strategies to recruit them for prevention, care and research Abstract Session, XVII International AIDS Conference, August 2008, TUAD0302

[B41] WHO, UNAIDS and UNICEF Towards universal access: scaling up priority HIV/AIDS interventions in the health sector. Progress Report 2008 2008 WHO

[B42] Mexico to Allow Drug Companies to Make, Sell Generic Antiretrovirals in Country, President Calderon Announces Kaiser HIV/AIDS Daily Report 2008 http://www.kaisernetwork.org/daily_reports/rep_index.cfm?hint=1&DR_ID=53722

[B43] Strathdee S Predictors of needle exchange programme utilization during its implementation and expansion in Tijuana, Mexico Abstract Session, XVII International AIDS Conference, August 2008, THAC0204

[B44] Saavedra J Sex between men Plenary Session, XVII International AIDS Conference, August 2008, TUPL0104

[B45] Nunez C Status of the response in Latin America Symposium, XVII International AIDS Conference, August 2008, TUSY1002

[B46] Reynaga E Sex Work Plenary Session, XVII International AIDS Conference, August 2008, WEPL0103

[B47] Carrillo H Se Fue al Gabacho: Mexico-US Migration and Vulnerability to HIV and AIDS Abstract Session. XVII International AIDS Conference, August 2008, TUAD02

[B48] Nilo A Key messages from the latest Latin American AIDS conference Symposium, XVII International AIDS Conference, August 2008, TUSY1003

[B49] Slater D Universal access in the Caribbean: what is the reality? Satellite, XVII International AIDS Conference, August 2008, SUSAT3101

[B50] Cenac V Structural Drivers of Stigma and Discrimination Satellite, XVII International AIDS Conference, August 2008, SUSAT3104

[B51] Paen C Lessons learned to roll back the infection rate in urban areas in Haiti: community-based responses Symposium, XVII International AIDS Conference, August 2008, THY0305

[B52] Caribbean public health authorities propose to eliminate vertical transmission of HIV and syphilis by 2015 Press Release 2008 Pan American Health Organization http://www.paho.org/english/dd/pin/pr080807b.htm

[B53] Figureoa P The HIV epidemic and the response in the Caribbean Symposium, XVII International AIDS Conference, August 2008, THSY0302

[B54] Pean C Regional conference strategy planning for response (CARICOM approach) Symposium, XVII International AIDS Conference, August 2008, THSY0305

[B55] Ivers LC Task-shifting in HIV care: a nurse-centered, community-based model of care in rural Haiti Abstract Session, XVII International AIDS Conference, August 2008 WEAX0103

[B56] Mukherjee J The intersection of access and adherence: improving retention in HIV programmes in resource-poor settings Poster Exhibition, XVII International AIDS Conference, August 2008, MOPE0146

[B57] Tawil O There is no single HIV Epidemic in MENA - learning from facts to shape the AIDS response in the region Symposium, XVII International AIDS Conference, August 2008, THSY0902

[B58] Lahouel N Part of the Solution - people living with HIV engaged as key actors to achieve Universal Access in MENA Symposium, XVII International AIDS Conference, August 2008, THSY0905

[B59] Karaouaoui A Keeping the focus: HIV prevention services for those most in need - perspectives from the field Symposium, XVII International AIDS Conference, August 2008, THSY0903

[B60] Kamarulzaman A Substance Use and Harm Reduction Plenary Session, XVII International AIDS Conference, August 2008, TUPL0103

[B61] Karkauri M Scaling up HIV testing: from an NGO perspective Poster Exhibition, XVII International AIDS Conference, August 2008, TUPE0967

[B62] Asouab F Morocco: Process and lessons learned from implementing harm reduction policy targeting injecting drug users in low HIV prevalence setting Poster Discussion, XVII International AIDS Conference, August 2008, WEPDE102

